# “Good fathers”: Community perceptions of idealized fatherhood and reported fathering behaviors in Mwanza, Tanzania

**DOI:** 10.1371/journal.pgph.0002587

**Published:** 2024-07-11

**Authors:** Alya Alsager, Juliet K. McCann, Alina Bhojani, Damas Joachim, Julieth Joseph, Andrew Gibbs, Mary Kabati, Joshua Jeong

**Affiliations:** 1 Department of Global Health and Population, Harvard T.H. Chan School of Public Health, Boston, Massachusetts, United States of America; 2 Department of Social and Behavioral Sciences, College of Public Health, Kuwait University, Kuwait City, Kuwait; 3 Hubert Department of Global Health, Rollins School of Public Health, Emory University, Atlanta, Georgia, United States of America; 4 Tanzania Home Economics Organization, Mwanza, Tanzania; 5 Faculty of Health and Life Sciences, Department of Psychology, University of Exeter, Exeter, United Kingdom; 6 Institute for Global Health, University College London, London, United Kingdom; 7 Gender and Health Research Institute, South African Medical Research Council, Pretoria, South Africa; 8 Centre for Rural Health, School of Nursing and Public Health, University of KwaZulu-Natal, Durban, South Africa; PLOS: Public Library of Science, UNITED STATES OF AMERICA

## Abstract

Globally, perceptions of idealized fatherhood have been expanding beyond men’s breadwinning roles to also value men’s engagement in nurturing care. While fathers’ caregiving behaviors are increasing, most childcare activities are still largely performed by mothers. In this study, we unpacked community members’ beliefs about the meaning of “good fathers” and explored the degree to which these values aligned with the main caregiving behaviors reported about fathers with young children under age 2 years in Mwanza, Tanzania. Qualitative data were collected as part of a broader formative research study for which we conducted in-depth interviews with 29 fathers, 23 mothers, 4 village leaders and 4 community health workers as well as 3 focus group discussions with fathers, 2 with mothers, and 6 with both fathers and mothers combined. For this secondary data analysis, we used a grounded theory approach combined with thematic content analysis to investigate the nature of fatherhood. We discovered four key ideals associated with “good fathers”: fathers as providers, nurturers, supportive partners, and authoritarians. The primary ideal of fathers as breadwinners was strongly aligned with the main reported practice of fathers trying hard to financially providing for their families. However, paternal behaviors reflecting ideals of fathers as nurturers and supportive partners were less practiced. Although ideals towards good fathers as authoritarian were least explicitly valued, many fathers were reported as engaging in controlling behaviors and using violence. The links between fatherhood ideals and behaviors was influenced by various factors, including poverty, men’s limited time availability at home, and restrictive gender norms. Overall, our results reveal some alignment but also inconsistencies between the ideal version of fatherhood and commonly reported paternal practices. These discrepancies highlight the need for further investigation into the underlying factors that both enable and constrain the links between fatherhood ideals and behaviors. Our study results have important implications for the design of interventions that seek to enhance fatherhood to improve the development and wellbeing of children and families.

## Introduction

The first years of life are a sensitive period of development that establishes the foundation for children’s later life course trajectories [1]. Ensuring that children receive nurturing care from all caregivers in the household is critical for supporting healthy early child development (ECD) [2]. Although much of the evidence on caregiving of young children has focused on women exclusively, there is growing evidence about the importance of men’s engagement too for ECD across low- and middle-income countries (LMICs) [3].

Multiple studies have documented how fathers’ caregiving practices and interactions with young children are associated with various ECD outcomes independent of the known contributions of mothers [4–6]. Fathers’ caregiving behaviors can encompass various types of activities, such as paternal play and communication, disciplinary practices, and involvement in other childcare and household activities (e.g., feeding children, preparing food, and cleaning the household) [7]. Fathers’ active engagement within families is not only beneficial for young children, but also associated with improved couples’ relationship outcomes, such as more equitable decision-making and reduced family violence, as well as improved maternal mental health [8–10]. Moreover, gender-transformative programs that sensitively engage both men and women to address harmful gender norms and relations while also promoting women’s empowerment, agency, and safety can improve both maternal and paternal caregiving and ultimately children’s health, nutrition, and developmental outcomes [11,12].

Various factors have been identified as shaping men’s engagement with young children in LMICs. Much of this literature has focused on socioeconomic and demographic determinants of men’s engagement. For example, studies have revealed that low paternal education, lack of employment, increased number of children, and poor family socioeconomic status are risk factors to paternal involvement [13,14]. However, in addition to individual and household sociodemographic characteristics, fatherhood is also importantly influenced by value systems and ideals [15–17].

Fatherhood ideals can broadly encompass attitudes (i.e., affective evaluations towards behaviors that can be observed at individual and community levels), beliefs, and norms (i.e., collective awareness and shared expectations or rules governing appropriate behavior within a group or context) [18,19]. In particular, attitudes and norms about fatherhood are fundamentally shaped by conceptualizations of masculinities, which refer to beliefs and practices around men’s roles and expectations [20,21]. In any given context there is a *hegemonic masculinity*, which is an idealized concept of what “men should be and do” [22]. Although this ideal is unobtainable for most men, hegemonic masculinity provides a template of aspiration through which men construct and make sense of their own masculinity [22].

Within heterosexual contexts, the hegemonic masculinity typically endorses and emphasizes men’s dominance and power over familial, social and economic structures and over alternative expressions of masculinity that are deemed “feminine” or less dominant [22,23]. In addition, it shapes spheres of manhood including parenting and fatherhood identities [23,24]. Typically, the hegemonic masculinity is a key driver of gender-divided roles within families–with fathers assuming primarily breadwinning roles outside the home [25], while women are responsible for domestic work and child-rearing [26,27]. This has resulted in a dominant ideal of fatherhood characterized in terms of men’s financial provisions for the child and household, which has been repeatedly underscored over the past decades in various contexts across sub-Saharan Africa [14,28,29]. Another common aspect of hegemonic masculinity in relation to fatherhood pertains to men’s power and control within families. These gender norms are reinforced by other social values such as family traditions and religious beliefs that legitimize men’s dominance over women and children and contribute to harmful behaviors such as the acceptability of men’s use of violence [29].

While fathers’ roles as the financially provider have been consistently prioritized across sub-Saharan Africa, more recent evidence has also suggested that fatherhood ideals are shifting to also value more nurturing forms of fathering such as active paternal engagement in caregiving and support in household chores [30–32]. Globally, sociodemographic transitions–such as women’s entry into the work force, increased urbanization, amalgamations in cultures and exposure to other belief systems–have contributed to evolving perceptions about positive fatherhood, and what an idealized father may do [20,33]. These changes in values and beliefs however are not uniform across sub-Saharan Africa, with many communities reluctant around male engagement in feeding or domestic chores driven by various contextual factors, such as poverty (e.g., economic pressures pushing fathers toward provider roles) [24], rural residency [34], and personal histories (e.g., upbringing and relationships with one’s own father either reinforcing traditional beliefs or prompting deviations from them) [35]. Thus, additional research unpacking the contemporary value systems that underpin perceptions of ideal fatherhood practices and expectations is needed across diverse cultural contexts.

Alongside these shifting ideals about fatherhood, fathers are also becoming increasingly more involved in caregiving behaviors particularly with children during the early years of life. Although women continue to assume the majority of caregiving roles, fathers are dedicating more time to nurturing care activities for their young children like play [36], communication [37], and feeding [38]. Additionally, studies have highlighted how fathers’ caregiving behaviors are interconnected and influenced by other household members, such as men’s coparenting relationships with their female partners and the roles of other family members, such as grandmothers and sibling children [32,39–41]. However, this evidence base on ideals of fatherhood and fathers’ caregiving practices has been largely disconnected from one another. Notable exceptions include research focused on teenage fathers from the United States [42,43] and a qualitative study from Uganda that found that despite fathers’ favorable attitudes and readiness to be more engaged in childcare, they experienced social pressure to conform to dominant forms of masculinity, which limited their actual involvement [44]. These studies however focused on particular individual-level characteristics (e.g., age) and did not comprehensively examine the social determinants shaping fatherhood ideals against those influencing fathering behaviors.

Therefore, the purpose of this analysis was to investigate ideals of “good fathers”; examine the extent to which these dominant ideals aligned with the main types of caregiving behaviors described of fathers; and identify the factors influencing both fatherhood ideals and behaviors in Mwanza, Tanzania. We conducted a secondary qualitative analysis using data collected as part of a formative research study that broadly aimed to explore fatherhood experiences through the diverse perspectives of fathers, mothers, community health workers, and community leaders. Understanding the contemporary values surrounding “good fathers” and its relationship with common practices of fathers can inform the design of social and behavior change interventions to support positive fatherhood in the Tanzanian context.

## Methods

### Study design

We used data from a larger phenomenological study aimed at describing the practices, barriers, and facilitators related to fathers’ caregiving in which participants were asked a series of broad questions related to parenting roles, mental health, and couples’ relationships [7]. In the present study, we further analyzed a subset of these data that focused on fathers’ caregiving behaviors and community perspectives surrounding “good fathers” to explore attitudes towards fatherhood and their alignment with fathers’ practices.

### Study site

This study was conducted in the peri-urban setting of Mwanza, Tanzania, located on the shores of Lake Victoria. The majority of household in Mwanza identify with the Sukuma ethic group in which lineage and inheritance typically follow patrilineal traditions and post-marital residency is patrilocal with women residing in her husband’s home or community [45]. Historically, the Sukuma have been cattle herders and farmers, which has shaped much of their social structure and cultural practices. The importance of cattle in Sukuma society historically influenced marriage and family structures, particularly through the practice of bridewealth (bride price), where livestock was a common form of payment. Although the prevalence of bridewealth has diminished, its historical influence on gender roles and expectations within households persists [46]. In present-day Mwanza, common income-generating activities include agriculture and small business ownership, with fishing also widespread in coastal communities [47]. Yet challenges such as climate change negatively affect economic opportunities [48]. This background has heightened the emphasis on the breadwinner model within families, where economic pressures have, in some cases, reinforced traditional gender roles [49].

### Sampling

Through collaboration between Tanzania Home Economics Organization (TAHEA) and government representatives, four study villages were selected for this study via a stratified sampling to ensure the inclusion of two coastal villages and two inland villages. In each study village, community leaders assembled a list of eligible caregivers and shared these lists with TAHEA. The research coordinator at TAHEA then randomly selected caregivers from these lists for participation in the study. Caregivers were eligible based on the following criteria: adult biological parent who was aged 18–65 years; had a child younger than 2 years of age; was in a relationship with the child’s other biological parent; and resided in the same house as partner and child at some point during the past month. We conducted in-depth interviews (IDIs) and focus group discussions (FGDs) with fathers and mothers. FGDs included fathers-only, mothers-only and mixed fathers- and mothers- groups. Specifically, mixed group discussions were included to encourage open dialogue between male and female participants, highlight any perspectives that may not have surfaced in father-only or mother-only discussions, and uncovered the particular areas of consensus and divergence in men’s and women’s viewpoints. These mixed groups also allowed use to triangulate findings from fathers-only and mothers-only FGDs. We also conducted IDIs with the community leader and community health worker (CHW) of each village.

### Data collection

Data were collected in June 2022 using topic guides developed by investigators at Harvard and the Tanzania Home Economics Organization. These guides were tailored for use in IDIs with fathers, IDIs with mothers, IDIs with community stakeholders, and FGDs with fathers alone, mothers alone, or fathers and mothers together. Topic guides comprised of semi-structured, open-ended questions that primarily focused on understanding parents’ roles and caregiving behaviors. For example, we asked: *“What does it mean to be a good father*?*”* and *“How does a good father care for and interact with his young child*?*”*. All interviews were conducted in Swahili.

Data were collected in a private setting of a centrally located landmark with the community (e.g., daycare center, village leader officer) by a team of five research assistants from Mwanza. Research assistants participated in a 7-day training led by researchers at Harvard University, which included three days of lecture-based instruction on study goals, early childhood development, parenting, men’s engagement, and qualitative data collection skills, and four days of piloting data collection tools and providing practice for data collectors in a non-study community.

IDIs were approximately 60–90 minutes while FGDs lasted approximately 90 minutes. The audio-recorded interviews were transcribed and translated into English by a team of translators from Tanzania, with a subset of approximately 15% of the transcripts selected at random to be checked for quality assurance. See elsewhere for more details on data collection procedures [7].

### Data analysis

Principles of grounded theory and thematic content analysis methodologies were applied to analyze data on community attitudes of “good fathers” and reported fathers’ caregiving practices. To analyze the behaviors of fathers, we ascribed pre-determined codes on common caregiving practices (e.g., play, communication, financially providing, care for nutrition, etc.) to participant responses about fathers’ routine behaviors. We then used principles of grounded theory by carrying out an inductive investigation into the ideals participants associated with engaged fathers and the relationship between these attitudes and fathers’ practiced behaviors. All de-identified English transcripts were independently coded by three research analysts (AA, JM, AB) using Atlas.ti 22 with 30% of these transcripts randomly selected for a second independent coding by another analyst. Weekly discussions with the research analysts were led by JJ to review findings and resolve any discrepancies and uncertainties in coding. Emerging themes were regularly discussed also with Tanzanian-based research collaborators to ensure our findings accurately represented the lived-experience of the community participants.

### Research team

The data collection was conducted by five research assistants from Mwanza. All held bachelor’s degrees, were bilingual in Kiswahili and English, and had previous experience in both early childhood research and qualitative data collection. The senior author led the study, had significant field experience in sub-Saharan Africa, and PhD-level experience in global public health, specifically in the areas of parental engagement, family well-being, and ECD. The research analysts were all masters-level graduate students at a university in the United States with prior research experience in ECD and family caregiving, prior field experience on ECD in East Africa, and experience with qualitative coding and analysis experience. The data analysis team consisted of international researchers from North America, East Asia, the Middle East, and East Africa.

### Ethical approvals

The research protocol for this qualitative study was reviewed and approved by the Institutional Review Boards of the Harvard T.H. Chan School of Public Health and the National Institute of Medical Research in Tanzania. Informed consent forms were read aloud by trained research assistants in Kiswahili. Participants were given opportunities to ask any questions during the consent process, and written informed consent was obtained from all participants. Additional information regarding the ethical, cultural, and scientific considerations specific to inclusivity in global research is included in the (S1 Checklist).

## Results

### Sample characteristics

We collected data from a total of 121 participants: 55 fathers, 58 mothers, and 8 community stakeholders. We conducted IDIs with 29 fathers, 23 mothers, 4 CHWs, and 4 community leaders, and 9 FGDs: 3 with fathers, 2 with mothers, and 4 mixed groups that combined both fathers and mothers. Between 4 to 10 participants were included in each FGD, with at least 2 mothers and 2 fathers represented in all mixed group discussions. Among community stakeholders, 6 were male and 2 were female. All caregivers were in heterosexual relationships. The average age of mothers and fathers was 27.0 and 36.1 years, respectively. Primary school was the highest level of education for most mothers and fathers (88% and 84%, respectively). Fathers were predominantly employed in agriculture (67%), fishing (25%), and daily labor (8%). All mothers were primarily responsible for household chores and childcare activities, and 83% also engaged in income-generating activities with 58% working in agriculture and the remainder in small businesses (e.g., selling sardines).

### “Good fathers” are providers

Fathers, mothers, and community stakeholders consistently perceived the defining characteristic of a “good father” was as a *provider*. Parents focused on fathers’ financial provisions and in relation to their children’s health and nutrition (e.g., purchasing of nutritious food for children). For example, one father shared, *“A good father is the one who meets family needs*. *Is food available*? *Are the clothes available*? *Is there a good place to sleep*? *A good father is the one who provides for the family as required*.*” (Fathers FGD*, *village #2)* This perspective was repeatedly expressed among community members to suggest a widespread belief that the ideal father was a breadwinner for the family. For instance, a community leader alluded to this norm by explaining how men who failed to provide would not be viewed favorably by others, “*A good father is the one who cares for his family*. *You cannot be called a good father without caring for your family–caring by providing food*, *clothing*, *providing an education*, *that is a good father*.*” (Community leader*, *IDI*, *village #2)*

When further probed to understand how important the role of provider was perceived relative to other characteristics, fathers and community stakeholders unanimously agreed that being a provider was the most important of all. This view was illustrated in the quote from the following father, *“The most important thing to me is providing food*, *clothes*, *home needs*. *These should be met and that is when you will be called a good father*.*” (Fathers FGD*, *village #1)* One father articulated fathers’ provider roles as fundamental to the family’s survival. He said, *“If you get sick*, *there is no money at home*. *Your families will be praying for your health not because they love you but because you have to get back to work and provide*.*” (Fathers FGD*, *village #1)* While nearly all fathers defined “good fathers” as providers, some mothers held differing opinions and believed that providing alone was insufficient. These mothers emphasized the importance of multiple characteristics in addition to providing, such as men’s active engagement in childcare and household responsibilities, which are presented in the following sections.

**Fig 1:**
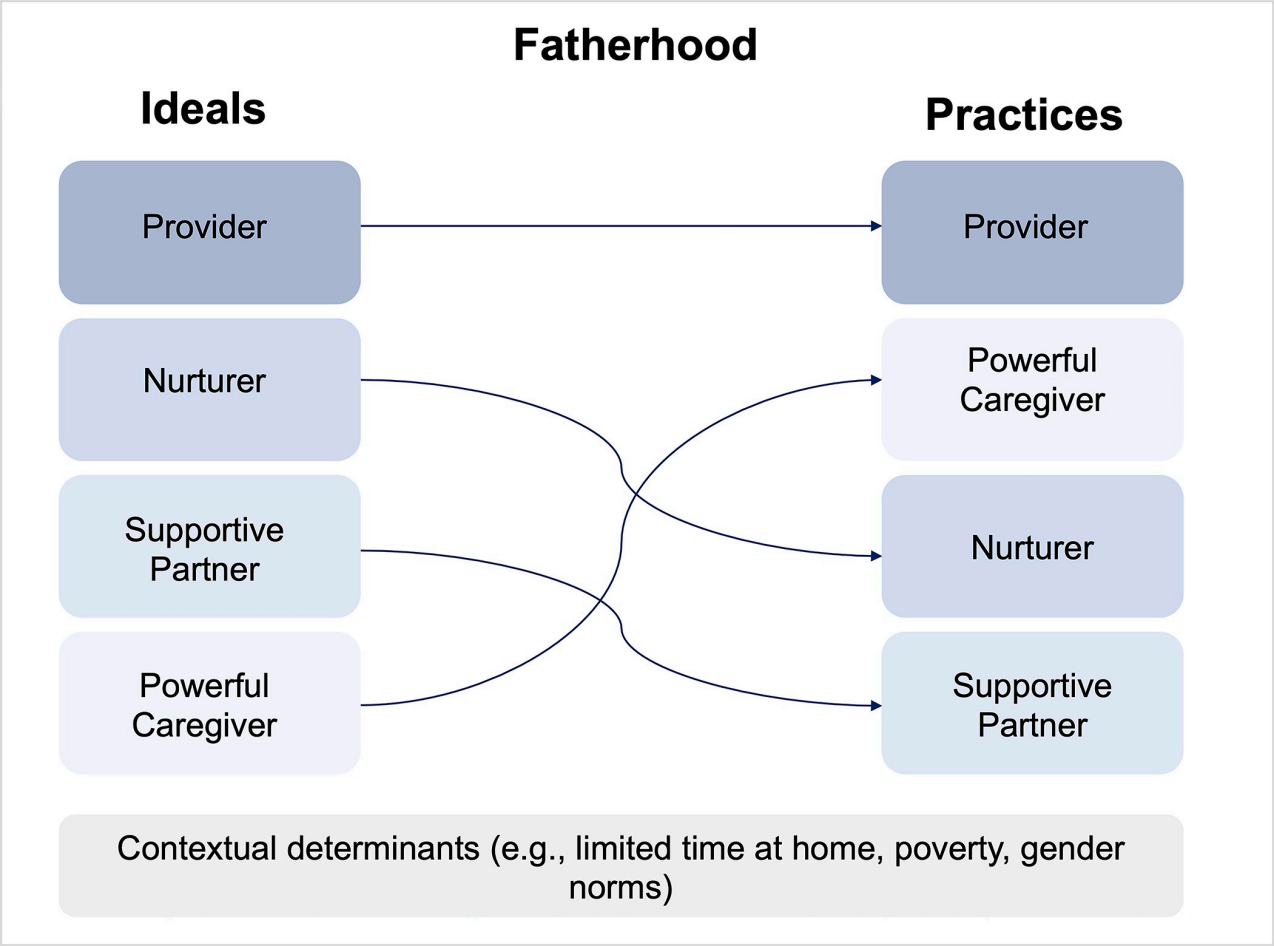
Overview of study findings regarding the relationship between fatherhood ideals and practices.

The dominant ideal of a “good father” as provider was strongly aligned with the main reported caregiving behavior of fathers in terms of providing for the family (Fig 1). All fathers described working to generate income for their families mostly through agriculture or fishing activities and typically from early morning to late evening. The majority of fathers and mothers shared how fathers provided various tangible goods for their child and family (e.g., food, clothes, healthcare-related expenses, small gifts). The strong value towards “good fathers” as providers and its link to financial provisions as the main paternal caregiving behavior was strongly shaped by community members’ experiences with poverty, food insecurity, and work instability. For example, one father shared, *“There are days when we struggle and sleep hungry and the family bears with us hoping God will bless us and we will have something to eat*.*” (Father #5*, *IDI*, *village #3)* In addition, fathers who were unable to provide perceived themselves as failing to achieve the dominant fatherhood ideal. For example, one father shared: “*They can’t be good fathers because they don’t have jobs*. *When they are asked to buy rice and they cannot*, *they are seen as not good fathers*.*” (Mixed FGD*, *village #5)*

The importance of fathers to provide was further reinforced by other sociocultural and gender norms. For example, when asked if there was anything that only fathers were able to do that mothers could not, one community leader mentioned fathers’ primary responsibilities in financial provisions and alluded to gender norms and the greater employment opportunities and financial access that were afforded to fathers, *“What only fathers do is providing*. *Mothers cannot provide*. *Most mothers here in the village do not have jobs*. *They are housewives so when you tell them to buy clothes and provide*, *where will they get the money*?*” (Community leader*, *IDI*, *village #2)* The value of fathers’ roles as providers was also intertwined with norms about men’s power. This sentiment was expressed widely by fathers and community stakeholders, as one father shared, *“Having a voice in the family depends on the one who is providing for the family*. *That is the one who has a voice*. *You cannot have a voice if your partner is the one who is feeding the family*.*” (Fathers FGD*, *village #2)*

The strong idealized notion of fathers primarily providing for families was even more clearly apparent when women discussed men failing to meet this role either through not providing adequately or consistently enough for their children and families. Mothers, some community stakeholders, and even a few fathers themselves discussed how fathers’ failure to financially provide equated to them not meeting expectations of the ideal father. For example, one father admitted that there were times he did not always use household finances in the best interest of the family such as for fulfilling family and child necessities, *“I am the head of the household but when I drink I will take the money that my wife has set aside and go to drink with it*. *That will cause problems*.*” (Father #5*, *IDI*, *village #3)*

### “Good fathers” are nurturers

The second most commonly held characteristic of a “good father" was in terms of nurturing the child. Respondents articulated this in terms of fathers’ physical closeness and direct interactions with children, such as fathers’ playing, carrying, and communicating with the child. One father emphasized, *“Both parents need to be involved in childcare*. *A father needs to care for his child whether a child’s mother is there or not*.*” (Mixed FGD*, *village #2)* This father went on to specifically note the importance of holding and playing with the child. All stakeholders–mothers, fathers, community stakeholders alike–highlighted the value of paternal engagement in stimulation activities and their physical proximity to children as a way for fathers to build stronger, more intimate relationships with their children, comfort their distressed child, and better understand their child’s needs. Mothers especially valued fathers’ engagement in caregiving activities and even more strongly than fathers and community stakeholders. For example, when asked to describe the qualities of “good fathers”, one mother shared, *“…to have time for the children like playing with the child*, *reading books for her*, *going to church together*. *This could build a bond between a father and the child*, *and the child will always be happy for the father*. *Even if she saw him far*, *she will run for him*.*” (Mothers FGD*, *village #1)* Similarly, one father explained, *“To me what is more important is to be close with the child to build an environment of love and closeness with the parents*. *By not showing love to the child*, *she/he can see you and run away with worry instead of being happy to see their father or mother*. *So to love her/him results in closeness*.*” (Fathers FGD*, *village #1)*

A few fathers identified specific factors as contributing to this emerging ideal of “good fathers” as nurturers. For example, one father remarked, "*Fathers didn’t have time for their children due to our work responsibilities and lack of knowledge on the importance of spending time with our children*. *But we are learning and seeing that it is very important to spend time with our children*. *We used to say that children are their mother’s responsibility*, *but we now understand that both parents are responsible for childcare*.*" (Mixed FGD*, *village #4)* Such sentiments highlight educational and normative barriers to men’s achievement of this ideal as well as the fact that this particular ideal of fatherhood has been changing.

While there was a dominant belief that men should have a nurturing role with young children, such paternal caregiving practices were not as commonly reported. Father engagement in play, communication, feeding, and child health promotion behaviors were not consistently performed by fathers with many describing challenges in doing so due to their busy schedules. For example, one father shared, *“If I had more money*, *I would get more time to spend with them*. *For example*, *if I decided to stay with my child all day*, *then I wouldn’t have money for his expenses*.*” (Father #3*, *IDI*, *village #2)* However, one mother suggested that even though fathers may not be home as much as women, there were still times when he could engage in these activities, *“Even when a father comes home late*, *he should try talking to his children in the morning to know what challenges they have been facing*.*” (Mixed FGD*, *village #1)* Additionally, mothers argued that, even when available at home, fathers did not always play with their children during their free time. Several respondents noted how restrictive gender norms hindered fathers’ engagement in such fathers’ nurturing behaviors, as one father noted, *“Others think that a good father is the one who carries a child a lot*. *That’s right but only that cannot look good on me to carry a child all the time and take a child to the clinic*.*” (Fathers FGD*, *village #2)* Similarly, one CHW highlighted how cultural norms deterred paternal engagement in nurturing care and maintained gendered divisions in childcare activities. This CHW shared, *“We have been brought up that way*. *Mothers stay at home to provide care for their children*. *A man cannot bathe or dress a child*. *I think that is just due to our culture*. *When dowry price is paid for women during marriage*, *it makes a man powerful*. *A woman is then supposed to do all household chores*.*” (CHW*, *IDI*, *village #1)*

Many respondents commonly expressed joint support for these two values of fathers as providers and nurturers and noted how both were important aspects of fatherhood. More specifically, several reiterated that fathers’ financial provisions was necessary but not sufficient, and that fathers needed to also engage in childcare activities in order to be regarded as “good fathers”. Although this dual role was endorsed by many respondents, some fathers highlighted a tension between these two values whereby they felt unable to engage in some of these more interactive parenting behaviors (e.g., play, holding their child, taking child out of the home) because they were also working to provide for their children, which they viewed as the most important of all. This view was also endorsed by a community leader, who considered other fathering behaviors secondary to providing, *“A good father is the one who does his roles*, *so it depends on what category of parenting that he does or doesn’t*. *Usually a father is the one who provides protection*, *food*, *shelter*, *If he does others are additional*, *they will depend with the age of a child*, *what should be done to a child depending on the age*.” *(Community leader*, *IDI*, *village #2)* Similarly, a few mothers also emphasized the importance of “good fathers” first financially providing, and then spending and playing with children with any spare time fathers had.

Although the majority of respondents described a rank ordering to these two dominant values, a few mothers provided an alternative perspective that these attributes do not have to be hierarchical with fathers first fulfilling their role as provider before then engaging childcare activities. For example, one mother shared, *“Even when he is far from home*, *he should call to talk with the baby*.*” (Mixed FGD*, *village #3)* Similarly, a few fathers believed that men did not necessarily have to provide financially in order to be considered “good fathers”. For example, one father shared, *“Being involved with your family makes a father to be good*. *You don’t necessarily need to have money in order for you to be a good father*.*” (Mixed FGD*, *village #6)* This father went on to explain men’s engagement in positive parenting and childcare activities was equally an important characteristic of a “good father”.

### “Good fathers” are supportive partners

The third dominant ideal of a “good father” and endorsed again by mothers, fathers, and community stakeholders was as a supportive partner. Respondents discussed this ideal in terms of positive couple’s relationship dynamics, such as through fathers’ inclusion of their partners in family decision making processes and joint engagement in childcare and household responsibilities. For example, one mother expressed the importance of communication and joint decision-making in her definition of "good fathers" as supportive partners. She shared, *“In my opinion*, *in issues concerning money*, *a good father must tell his wife about his income and together they should plan how they will take care of their family*.*” (Mixed FGD*, *village #1)* Multiple mothers and fathers idealized fathers’ positive involvement using specific terms such as “helping”, “assisting”, and “supporting” their partners. For example, one mother described a good father as one who “helps a mother” with childcare and further went on to endorse the value of fathers’ nurturing caregiver and capacity to independently respond to the child’s needs, “*A responsible father helps a mother with parenting so she can continue with other house chores*. *Even if a child is crying*, *he can manage to calm her down so the child can stop crying*. *Also a father can realize if a child is crying for hungry or what reason*. *That is how a responsible father is in our community*.*” (Mothers FGD*, *village #1)* As another example, while one father similarly valued men’s engagement in childcare activities and partner support as dimensions of ideal fatherhood, he also underscored the tension with restrictive gender norms that assigned such activities as women’s responsibilities:

“*Other fathers will see you as being controlled by your wife since you are doing what she is supposed to do*. *But for me*, *I see the mother’s and father’s roles are the same*, *since there is a time when a mother can become overloaded by house works and the child is supposed to be given porridge*, *so you have to take the child and give her porridge so as the mother can continue with other works*. *Although other men will see you as you are out of your mind*, *but to my side I see it’s better to help each other*.*” (Fathers FGD*, *village #1)*

In addition to childcare activities, several parents mentioned that supportive partners also participate in and share household responsibilities with their partners. For example, one father remarked, *“And being a good father is working together with your family and sharing household chores*. *For example when a mother is washing clothes you can light a fire or wash dishes*. *And you will hear people say you love each other*.*” (Fathers FGD*, *village #1)* This example highlights this father’s belief that men who act in this way are viewed positively by others and again showcasing how social norms shape fatherhood value systems. Lastly, a few parents also valued other related characteristics such as “good fathers” being kind and respectful to their partners. One father shared, *“Being a good father is not only about providing*. *If you provide all that is needed at home*, *but you come home drunk and disturb your family that is not good*. *You must be kind*. *When you come home children get excited*. *Even if you leave home a lot of money but are not peaceful*, *that is not good*.*” (Fathers FGD*, *village #1)*

However, personal accounts of fathers engaging in these styles as supportive partners was not as commonly reported as this ideal itself. In particular, men’s involvement in childcare and household activities was quite rare and limited to extenuating circumstances whenever mothers were not available to do these tasks. For example, one father shared:

“*We help our wives when they are sick*. *We can even take them to the hospital but it is difficult for us to do chores like washing the dishes or bathing the children because we will be laughed at; it is a shame for a man like me to be seen doing these chores*. *People will think that I don’t have a voice in my family*. *I can help to carry a child when he/she is crying while his/her mother is busy*, *but I’ll hand him/her to his/her mother when she is done with the chores*.*” (Mixed FGD*, *village #4)*

Similarly, family decisions were not commonly made jointly between partners, especially as reported by mothers and specifically regarding financial decisions. For example, when one mother was probed on why she believed her partner was the primary decision-maker, she responded, *"because he is the one who owns everything at home*, *he has the final say in decision making*.*” (Mother #1*, *IDI*, *village #2)* The majority of fathers, mothers, and community stakeholders alike reported that “final” decisions were always made by fathers.

This misalignment was predominantly due to restrictive gender norms, cultural traditions, and fathers’ limited time spent at home. For example, when probed why he does not partake in household chores, one father explained in terms of gender norms, *“She does those chores because she is a woman*. *A man gets married in order to get someone to help him with the household chores*.*” (Father #1*, *IDI*, *village #4)* Moreover, some fathers feared that if they supported their partners in household chores or childcare, they would appear to be weak, bewitched, or controlled by their wives. This view was expressed repeatedly by many community members. For example, one community leader shared, *“For example*, *if a mother is in bed and children need to eat*, *then a father will cook*. *It is not like they cannot do such tasks*, *they fear what the community will think of them taking on mothers tasks*. *That is what leads to mothers taking all the roles*.*” (Community leader*, *IDI*, *village #4)* Another example comes from a mother who noted this as well:

“*When a man has a wife*, *he feels embarrassed to do such tasks*. *He thinks*, *‘What if people see me doing this?’ Most of them cannot do that*, *even when a woman is sick they would tell you to wake up and cook for children*. *If you ask for their help*, *they would say that they are leaving for work*, *but in fact they don’t want to do those activities*.*” (Mixed FGD*, *village #1)*

A few mothers and fathers challenged the idealized role of fathers as being supportive partners and noted how cultural traditions was a particular barrier. For example, one mother shared:

"*The majority of society is of Sukuma tribe*. *So for men from Sukuma tribe*, *it is difficult to do women’s work*. *If you find your husband helping you like washing the dishes he will say that he has been bewitched*. *So it is the tradition that men are not supposed to do women’s work*. *So the male parents leave it to us female parents to do the female work*, *that is*, *there is a separation of work*. *Cooking*, *bathing the child*, *work to do*, *just do it*.*" (Mixed FGD*, *village #4)*

Although many fathers mentioned instrumental partner support as a marker of a "good father", some fathers explained that at the end of the day they would be chastised by peers as “being ruled by my wife” if seen engaging in household chores like cooking. This fear of being perceived as controlled or “bewitched” deterred some fathers from engaging in tasks or roles traditionally seen as "feminine." This tension was underscored in an exchange with a community health worker:

“*CHW: Partners can help each other with chores*. *You can find some men washing clothes*, *cooking or carrying a child*.

*Interviewer*: *Why do you say that some of them do those tasks?*

*CHW*: *It is because others do not do these tasks*.

*Interviewer*: *Why?*

*CHW*: *In the village environment*, *many of the fathers do not do these tasks*. *When neighbors see him washing clothes they would say*, *‘I have seen this father washing clothes*, *he is suffering*.*’” (CHW*, *IDI*, *village #2)*

Lastly, fathers also reported that time spent working limited their availability to assist their partners in family activities. One father expressed how working long hours impacted his temperament and subsequently his ability to be both a nurturer and a good partner, *“A man has to go to work from 6 am to 8pm and they come home tired*. *You cannot get time to go near a child or when a child comes near you*, *you act unfriendly because you are tired*. *Even the communication with the wife becomes poor*, *you are angry all the time*.*” (Fathers FGD*, *village #2)*

On the other hand, fathers who regularly demonstrated actions of supportive partners explained that this was because they were “educated” and were cognizant of the how this benefited their partners and overall family. For example, one community health worker shared, *“Being a good father depends on the kind of education the father has*. *This will make him be close to his family*. *They give each other advice*, *discuss things together*, *agree on the same issues such as farming*.*” (CHW*, *IDI*, *village #4)* However, it was not clear what type of education they were referring to or from whom or how they received this education. Nevertheless, a few fathers further articulated knowledge about the positive effects of men’s instrumental support, *“From the beginning*, *it was difficult for fathers to wash dishes or wash clothes because we were not educated about this*. *We oppressed our wives with all the chores but it is different now since men/fathers are able to help with some chores*. *We have learnt that there are no chores specifically for women*. *We participate in doing all chores at home*.*” (Mixed FGD*, *village #4)*

### “Good fathers” are authoritarian caregivers

The fourth and final ideal that emerged was the perception of "good fathers" as inherently authoritarian figures. Although this ideal was the least commonly expressed and explicitly mentioned by only a few of fathers, this minority group of parents expressed strong beliefs about men’s power in directing, protecting, disciplining, and making decisions largely independently over their partners, children, and families. For example, in an FGD, a father and mother underscored the importance of fathers being strict:

“*Father: A good father is not too humble*. *He is supposed to be firm and make hard decisions for his family*.

*Mother*: *When a father is too humble*, *even his child would not listen to him*.*” (Mixed FGD*, *village #3)*

Again fathers referred to gender norms and societal expectations to explain the value underlying men’s control over their families. For example, one father highlighted how men are expected to make all the household decisions and when probed about how he felt, the father replied, *“[I feel] good because that is how it is*. *We have grown with that system*. *We have grown up seeing our father doing that*.*” (Fathers FGD*, *village #3)*

In addition, when asked whether there was anything unique about the role of fathers compared to mothers, both fathers and mothers suggested fathers had greater power both socially but also physically. This manifested in community members’ perceptions about parental discipline such as the description of fathers using more severe forms of violence (e.g., beating, slapping) and the belief that fathers’ discipline was more effective than that of mothers. For example, one father shared: *“There are few things we as fathers can do and mothers cannot do*, *for example punishing the child when they did something wrong*. *Mothers have soft hearts*, *they cannot manage to do that*.*” (Father #6*, *IDI*, *village #4)* Another father expressed a similar sentiment, “*A mother may not discipline a child as well as a father does*. *For example*, *when a child wets a bed*, *the child’s mother may not discipline him/her*. *But when the father beats the child*, *then he/she won’t repeat bed wetting*.*” (Fathers FGD*, *village #4)* This value towards fathers as disciplinarians was also endorsed by several community leaders and shaped again by gender norms, *“If a child plays with a radio at home and damages it*, *the father will use a harsh voice and sometimes punish the child*. *However*, *the mother uses words to teach and in a good language*, *like*, *‘Don’t do that again*. *If you do that again I will tell your father and he will whip you*.*’ Fathers command children to follow order so they cannot do that again*.*” (Community leader*, *village #1)* Relatively fewer mothers mentioned ideals related to power in their characterizations of “good fathers.”

Although authoritarian ideals appeared to be the least outwardly valued among community members, such authoritarian behaviors were reported frequently across households and in the community. Fathers’ dominance and exertion of power in family relationships were endorsed through reported violence against partners and children, exclusive decision making, and harsh communication styles with their partners. Fathers’ engagement in such behaviors were again rooted in patriarchal gender norms. For instance, one father shared, “*I am the head of the family*. *I have the last say on what should be done in the family*. *I’d feel ashamed if she had a greater voice than me in the family*. *In our community*, *a man is supposed to have a greater voice in his family*. *I would feel humiliated to my neighbors and friends*. *Children would not respect me as their father knowing that their mother has a greater voice*.*” (Father #2*, *IDI*, *village #2)* Another father highlighted how religious beliefs also play a role in shaping beliefs around power and decision making, *“As I said from the beginning a father is the head of the family*. *It is impossible to have equal positions between a couple*. *Even God created us in that way*. *When a father loses his voice in the family it leads to quarrelling*. *So fathers should be the ultimate decision makers*. *A wife can provide advice but the decision should come from a father*.*” (Mixed FGD*, *village #1)*

Even though the majority did not explicitly value authoritarian styles of fatherhood, still many fathers alluded to the acceptability of these styles and even described such approaches as necessary in times of family conflict, which reinforced the deep-rooted expectation and norm of women being submissive, and the justification of violence in cases where they are not. These gender norms pertaining to the acceptability of violence can be seen in the following example shared by one father, *“No*, *but women are beaten because they have not been submissive*. *Who has to be submissive between a man and a woman*? *A man doesn’t go down*. *You may be talking slowly and she talks loudly for all the neighbors to hear and that’s when a man gets angry and punishes a woman*.*” (Fathers FGD*, *village #2)* Additionally, fathers validated their harsh behaviors against children and mothers by referencing cultural and generational norms surrounding power, masculinities, and the expected subordination of women. For example, one father noted, *“I have the power to slap my partner when she has done something wrong*. *It is because she is my wife*, *I took her from her parents’ home and I brought her to my home*. *My wife and children have to listen to me*.*” (Mixed FGD*, *village #3)*

## Discussion

In this qualitative analysis, we explored the primary ideals of fatherhood and the alignments with the main reported caregiving practices of fathers in Mwanza, Tanzania. We found 4 distinct ideals about “good fathers”: fathers as providers, nurturers, supportive partners, and as authoritarian caregivers. Of these, the dominant ideal was as breadwinners of the family while values about fathers being powerful were the least explicitly articulated. Nevertheless, we uncovered a spectrum of community members’ perceptions surrounding fatherhood ideals and identified various underlying determinants of fatherhood attitudes and practices in the Tanzanian context.

Prior studies regarding the ideals of fatherhood have mostly focused on the value that communities place on fathers’ roles as the breadwinner, as driven largely by societal expectations, gender norms, and poverty [14,19,20,24]. Our study confirmed that the expected role of fathers as providers was the dominant ideal held by community members in Mwanza, Tanzania. This belief strongly shaped why financial provisions was the primary reported caregiving behavior of men. Although this was true for the majority of respondents, some participants on the other hand noted how not all fathers carried out these ideals in practice due to financial hardships and poverty. We also discovered several other factors as challenging fathers from financially providing, including excessive alcohol consumption and marital challenges, which have been noted in other studies from similar settings [14,50].

Following the ideal of “good fathers” as providers, community members expressed the importance of “good fathers” as nurturing, engaged, and intimately involved with their children. Increasingly, studies have highlighted fathers’ growing interest and desire to engage more in nurturing caregiving practices [33,36], for example with national survey data in LMICs revealing men’s are becoming more engaged in stimulation activities with children [4]. At the same time, gender inequalities in nurturing caregiving practices persist across sub-Saharan Africa with mothers still disproportionately engaging in the vast majority of childcare activities by herself [14,51]. As described in our study and others, limited father engagement in play and communication appeared to be primarily because men reported spending most of their day out of the home and away from the child due to work and in order to fulfill their ideal of fathers as providers [36,52]. However, our results did not suggest that fathers’ limited engagement in childcare was necessarily due to ideals that discounted or devalued such behaviors. Indeed, other recent studies from South Africa and Uganda, for example, have also highlighted how mothers and fathers alike value childcare activities such as play and communication and view these behaviors as ways to develop strong early bonds with their child [36,44].

Similarly, fathers’ role as supportive partners is increasingly being recognized as a critical aspect in recent conceptualizations of fatherhood [40,44,51]. We found that the majority of mothers and community stakeholders defined good fathers as those who were supportive of their partners in childcare activities, household chores, and the decision-making process. However, fewer fathers reported engaging in activities that were consistent with these ideals. Some men perceived stigma surrounding their participation in household chores and shared caregiving and expressed fear of being viewed as weak, controlled, or "bewitched" by their wives. This social pressure and fear of judgment deterred many from moving beyond traditionally defined gender roles and responsibilities [32]. Notwithstanding, there were some exceptions, with some fathers attributing awareness of the benefits of men’s engagement and positive community perceptions (e.g., being seen as loving their partners) as motivating factors for support their wives in various roles. Given that ideals of community stakeholders differed considerably from fathers’ ideals towards partner support, our results highlight the importance of strategically harnessing these community perceptions to transform the fathers’ ideals towards gender equality, which may in turn enable fathers to adopt behaviors that are supportive of their partners [11].

The final ideal, which was mentioned relatively the least among community members, pertained to “good fathers” as being authoritarians. Although this fatherhood ideal was not explicitly perceived as a central value, most respondents reported fathers’ engagement in such authoritarian and aggressive behaviors in their everyday lives. For instance, fathers’ harsh disciplinary practices were frequently noted in addition to men’s perpetration of violence towards their partners. Key drivers of these behaviors of fathers appeared to be rooted in patriarchal norms and ideals of male dominance, power, and control over women and broader social norms that perpetuate the acceptability of these actions [29,44,53]. Our findings suggest that while some fathers may not have idealized power-related behaviors as defining good fatherhood, many justified such behaviors as acceptable, especially when confronted with perceived "insubordination" from women. Therefore, even if not seen as an ideal characteristic of fatherhood, there’s a belief that it’s a required approach for fathers to maintain authority. More generally, this ideal may not have been as explicitly salient perhaps due to the strong influence of deeply rooted authoritarian norms of the cultural context [14], and given the high prevalence of IPV in Mwanza, with nearly half of women reporting experiences of physical IPV [54]. At the same time, it is also plausible that authoritarian ideals did not emerge about fathers because such views are becoming increasingly less acceptable in many societies, including Tanzania [55]. Nevertheless, fathers’ use of authoritarian caregiving practices were still commonly reported.

Overall, our findings revealed a complex relationship between community ideals of fatherhood and the caregiving behaviors reported about fathers, whereby for some themes such as financial provisions the connection was apparent but other values did not translate as strongly to paternal actions. Even though some men endorsed gender egalitarian ideals towards caregiving (i.e., good fathers as nurturers and supportive partners), they did not necessarily practice the positive caregiving behaviors associated with these ideals. Previous research that found that shifts toward more gender egalitarian ideals were not always aligned and in some cases were contradictory with men’s actual engagement in caregiving [24,56,57]. For example, a study on the roles of men and women in maternal and child nutrition in urban South Africa found that although men expressed a desire to be more involved in their child’s nutrition, traditional gender roles around food preparation as a “feminized” task and lifestyle choices like alcohol and tobacco consumption deterred their participation. We extend this prior evidence by identifying a range of other contextual factors–such as knowledge gaps (e.g., why play is important for ECD), poverty, limited opportunities for fathers to spend time with their children during the day, and broader sociocultural and gender norms–that interact and attenuate links between fatherhood values and practices.

Another key finding worth noting is how these various types of fatherhood ideals and behaviors are not mutually exclusive. For example, men who were supportive partners and nurturing caregivers still valued their role as financial provider, however their identities and reported fathering behaviors were discussed more holistically to encompass a broader range of actions and support (e.g., financial, interpersonal, emotional, childcare). These results highlight how fatherhood programs should target these multiple fatherhood values simultaneously and in a comprehensive manner rather than focusing on one dominant norm in isolation.

Fatherhood interventions that address restrictive gender attitudes and social norms surrounding paternal caregiving are increasingly being implemented in LMICs [58]. For example, evaluations in Uganda, Rwanda, and Tanzania found that gender-transformative fatherhood interventions that redefine masculinities and challenging men’s ideas of power and control can effectively improve gender equitable attitudes and fathers’ positive behaviors like reducing child maltreatment and IPV, increasing paternal time engagement in domestic chores, and maternal decision-making power [11,59,60]. Moreover, several programs have recognized the unique opportunity for addressing gender attitudes and masculinities during a father’s transition into parenthood that can be a formative time for influencing men’s caregiving behaviors and setting strong father-child relationships that carry forward later in life [61,62]. To address these value systems and norms surrounding fatherhood, noteworthy interventions have used strategies such as community campaigns, engaging influential leaders, and fostering open discussions in the community that have shown promise in changing community ideals on fatherhood in various LMICs [58,59]. Moreover, in terms of program content, successful fatherhood norms change interventions have directly incorporated education and reflection about masculinities to challenge men’s existing gender beliefs and foster a deeper understanding of equitable gender norms, which have been effective for increasing men’s engagement in household and childcare activities and reducing violence [11,44,59].

At the same time and recognizing again how fatherhood was primarily idealized in terms of men’s financial contributions, fatherhood interventions should not discount or ignore the value men and women place on fathers’ breadwinning roles. Several prior men’s engagement programs have faced challenges with low fathers’ participation rates and documented this was due to men’s greater priority towards seeking employment opportunities and fulfilling their role as providers [58,63]. These program evaluations highlight the tensions that fathers face in their value systems but also the structural conditions of extreme poverty that challenge fatherhood. Although a few livelihood support programs have included fathers [64,65], programs to date have not been designed in a concerned manner to provide both economic strengthening and fatherhood support from a social and behavior change perspective to promote positive caregiving among male caregivers of young children in LMICs. Thus, recognizing the main perceived identities held by men as financial providers and incorporating economic support within men’s engagement interventions so that men can fulfill both their financial and nurturing caregiving responsibilities may potentially be more effective than programs targeting men’s engagement in childcare alone.

Several limitations of this study should be noted. First, the results of this study come from a secondary analysis with a limited set of questions about perceived values regarding fatherhood. We did not specifically unpack distinctions between attitudes and norms or explicitly ask about the perceived relationships between attitudes, norms, and behaviors. Future research should further clarify these nuances and differentiate among the various types of norms (e.g., descriptive, injunctive norms) in order to better understand the pathways by which these ideals and perceptions influence behaviors. Although we’ve provided an initial picture of this relationship, the underlying mechanisms and processes require more detailed examination. Second, as with any qualitative investigation based on interviews, it is difficult to determine whether fathers indeed engaged in the caregiving behaviors that they reported as doing. We sought to triangulate these reports about fathers’ practices by incorporating the perspectives of mothers and community stakeholders to obtain more representative experiences. Finally, our findings are specific to peri-urban Mwanza, and therefore, ideals towards fatherhood and fathers’ caregiving behaviors may be different if the study was conducted in other contexts within Tanzania or elsewhere.

## Conclusions

Over the past decade, ideals and values surrounding fatherhood have been shifting across LMICs to broaden beyond hegemonic masculine ideologies of fathers as providers and authoritarians to also value more engaged fathering roles, including as nurturers and supportive partners. Despite these evolving values, such ideals are not necessarily translating to men’s increased engagement in these nurturing care behaviors with their partners and young children. Our study fills a gap in the fatherhood literature by substantiating this broad range of contemporary fatherhood ideals in the Tanzania context and providing insight into the myriad factors that influence perceptions of “good fathers” and their associations with fathering behaviors. Better understanding how ideals shape practices and the factors that enable or constrain their links can guide the design of improved fatherhood interventions to increase paternal caregiving behaviors and ultimately improve the wellbeing of children and families.

## Supporting information

S1 Checklist Inclusivity in global research. (DOCX)
